# Fragilities Caused by Dosage Imbalance in Regulation of the Budding Yeast Cell Cycle

**DOI:** 10.1371/journal.pgen.1000919

**Published:** 2010-04-22

**Authors:** Kazunari Kaizu, Hisao Moriya, Hiroaki Kitano

**Affiliations:** 1 Department of Science and Technology, Keio University, Yokohama-shi, Kanagawa, Japan; 2 Sony Computer Science Laboratories, Shinagawa-ku, Tokyo, Japan; 3 The Systems Biology Institute, Minato-ku, Tokyo, Japan; 4 Research Core for Interdisciplinary Sciences, Okayama University, Okayama-shi, Okayama, Japan; 5 PRESTO, Japan Science and Technology Agency, Chiyoda-ku, Tokyo, Japan; 6 Open Biology Unit, Okinawa Institute of Science and Technology, Kunigami, Okinawa, Japan; National Institute of Diabetes and Digestive and Kidney Diseases, United States of America

## Abstract

Cells can maintain their functions despite fluctuations in intracellular parameters, such as protein activities and gene expression levels. This commonly observed biological property of cells is called robustness. On the other hand, these parameters have different limitations, each reflecting the property of the subsystem containing the parameter. The budding yeast cell cycle is quite fragile upon overexpression of *CDC14*, but is robust upon overexpression of *ESP1*. The gene products of both *CDC14* and *ESP1* are regulated by 1∶1 binding with their inhibitors (Net1 and Pds1), and a mathematical model predicts the extreme fragility of the cell cycle upon overexpression of *CDC14* and *ESP1* caused by dosage imbalance between these genes. However, it has not been experimentally shown that dosage imbalance causes fragility of the cell cycle. In this study, we measured the quantitative genetic interactions of these genes by performing combinatorial “genetic tug-of-war” experiments. We first showed experimental evidence that dosage imbalance between *CDC14* and *NET1* causes fragility. We also showed that fragility arising from dosage imbalance between *ESP1* and *PDS1* is masked by *CDH1* and *CLB2*. The masking function of *CLB2* was stabilization of Pds1 by its phosphorylation. We finally modified Chen's model according to our findings. We thus propose that dosage imbalance causes fragility in biological systems.

## Author Summary

Normal cell functioning is dependent on balance between protein interactions and gene regulations. Although the balance is often perturbed by environmental changes, mutations, and noise in biochemical reactions, cellular systems can maintain their function despite these perturbations. This property of cells, called robustness, is now considered to be a design principle of biological systems and has become a central theme for systems biology. We previously developed an experimental method designated “genetic tug-of-war,” in which we assessed the robustness of cellular systems upon overexpression of certain genes, especially that of the budding yeast cell cycle. Although the yeast cell cycle can be maintained despite significant overexpression of most genes within the system, the cell cycle halts upon just two-fold overexpression of M phase phosphatase *CDC14*. In this study, we experimentally showed that this fragility is caused by dosage imbalance between *CDC14* and *NET1*. Interestingly, fragility of regulation of separase gene *ESP1*, potentially caused by dosage imbalance, was masked by regulation of other factors such as *CDH1* and *CLB2*. We thus propose that dosage imbalance causes fragility in biological systems.

## Introduction

Intracellular biochemical parameters, such as gene expression levels and protein activities, are highly optimized in order to maximize the performance of biological systems [1]–[4]. On the other hand, these parameters operate within certain limitations to maintain the function of the system against perturbations such as environmental changes, mutations, and noise in biochemical reactions. This robustness against fluctuations in parameters is considered a common design principle of biological systems [5]–[7].

The cell cycle is a series of events that leads to cellular duplication, and the regulatory system is highly sophisticated to precisely maintain cellular integrity [8]. The budding yeast *Saccharomyces cerevisiae* is an excellent model organism to understand the principle of cell cycle regulation because of its ease in use with molecular genetic techniques. Cell cycle regulation has been integrated into a mathematical model called Chen's model [9]. This model implements interactions of about 25 genes involved in the budding yeast cell cycle to reproduce over 100 mutant phenotypes, and thus, has become a standard for measuring the robustness of the budding yeast cell cycle [10]–[12].

**Figure 1:**
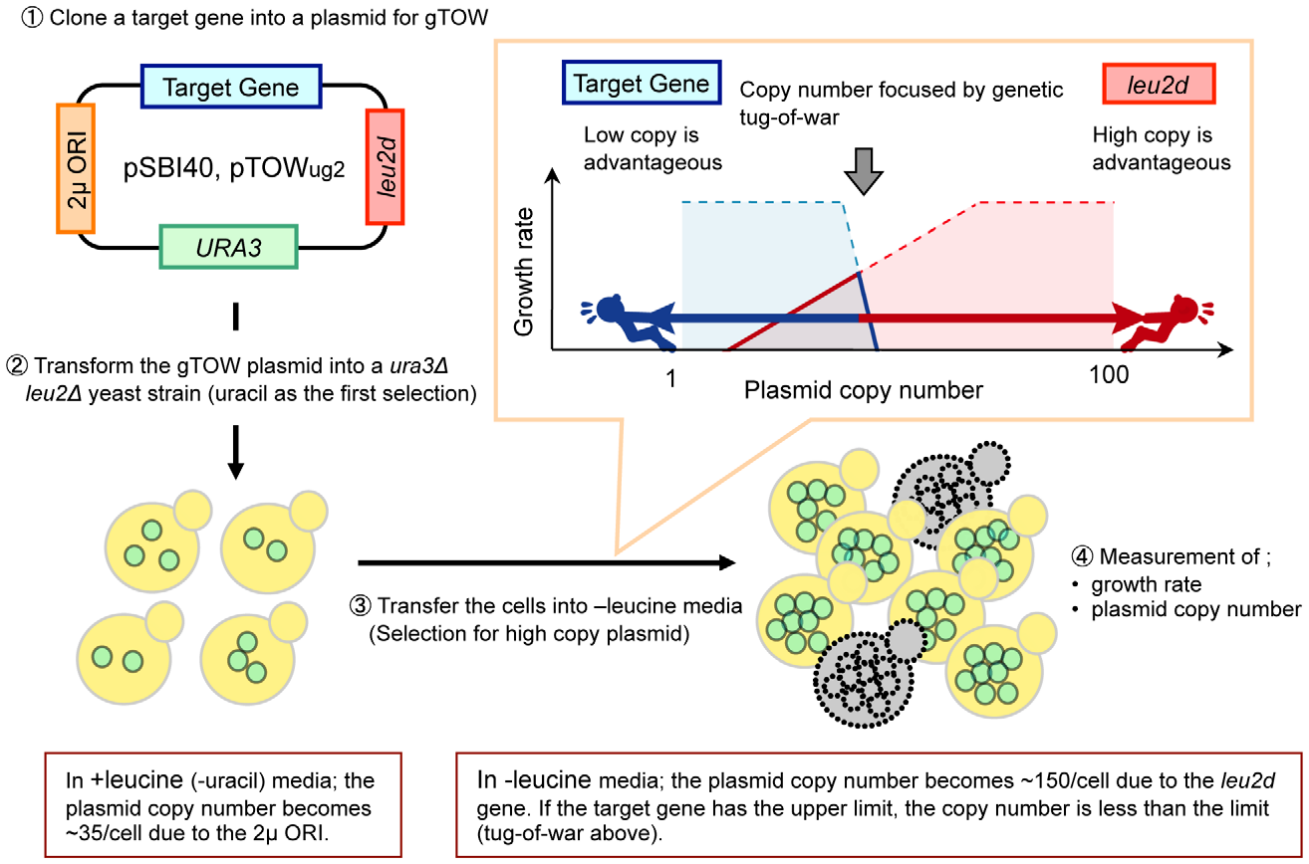
Schematic representation of the genetic tug-of-war (gTOW) experiment. gTOW is an experimental method with which upper limit of copy number of certain target genes can be determined. Details of the experiment are as described previously [13].

The robustness of a cellular system can be assessed by perturbation analysis of the extent to which each intracellular parameter can be changed without disrupting the function of the system [1]. To assess the robustness of the budding yeast cell cycle, we used a previously developed genetic experiment designated “genetic tug-of-war” (gTOW) to measure the copy number limit of overexpression of certain target genes [13]. In gTOW, a target gene with its native promoter is cloned into a special plasmid, and the plasmid copy number can be increased just before cell death (Figure 1) [13]. In this method, the copy number limit of gene overexpression is measured as a fold increase and compared with its native expression level.

Using gTOW, we measured the copy number limit of overexpression of 30 cell cycle-related genes that varied from <2 to >100 [13]. Although these numbers are thought to reflect the robustness of the subsystems harboring these genes, it is not easy to identify the molecular mechanism behind the phenomenon causing the variation because robustness arises from interactions between multiple components of the system. Analysis using mathematical models helps to identify the mechanism responsible for the robustness of biological systems [1], [14]. We compared the gTOW data with Chen's model and discussed the mechanisms underlying fragility and robustness of the yeast cell cycle in response to overexpression of several genes [13].

In this study, we define a cellular system has robustness if its normal mode of operation is hardly destroyed even when amount of a certain component in the system largely fluctuates. And we define a cellular system has fragility if its normal mode of operation is easily destroyed when amount of a certain component fluctuates. In this study, “fluctuation of component” corresponds to the increase of gene copy number in the cell, and the increase of gene expression parameter in the computer simulation (both manipulations cause gene overexpression). When the cell is viable despite overexpression of a certain gene, we call that the cellular system has robustness upon overexpression of the gene. When the cell is not viable due to minor overexpression of a certain gene, we call that the cellular system has fragility upon overexpression of the gene.

**Figure 2:**
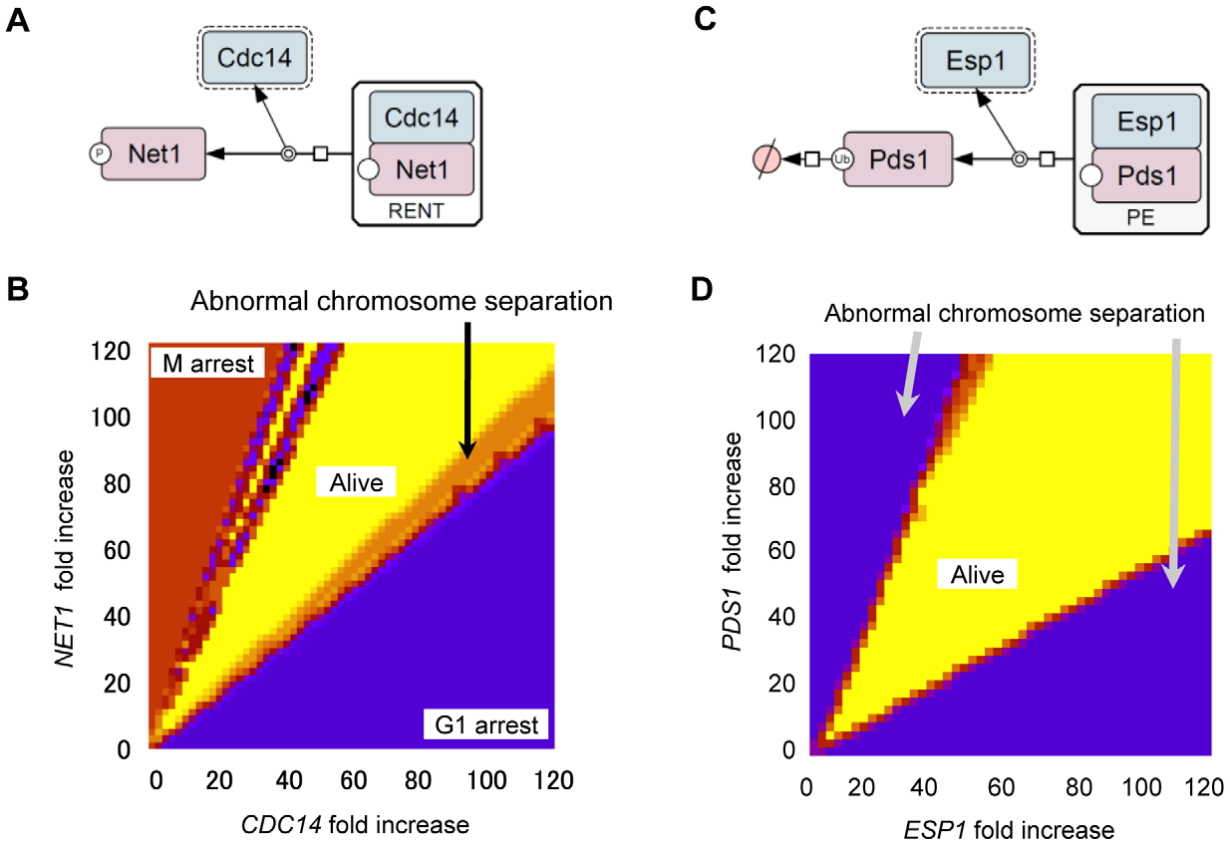
Computational analysis of quantitative relationship between *CDC14* and *NET1* as well as *ESP1* and *PDS1*. Diagrams representing Cdc14 (A) and Esp1 (C) regulation by 1∶1 binding with their inhibitors Net1 or Pds1. The molecular interactions were described with previously described graphical notations using CellDesigner4.0 software [50], [51] (A,D). (B,D) Results of two parameter viability test of Chen's model. The expression levels of *CDC14* and *NET1* (B) and *ESP1* and *PDS1* (D), were systematically changed. In (B), *x*-axis is the fold increase in the *CDC14* transcription rate (k_s,14_) and *y*-axis is for that in the *NET1* transcription rate (k_s,net_). In (D), *x*-axis is the fold increase in total concentration of *ESP1* ([Esp1]_T_) and *y*-axis is for that in the *PDS1* transcription rate (k′_s,pds_, k″_s1,pds_ and k″_s2,pds_). The viability test was performed using each parameter set (detail of the viability test is described in Text S1 and Figure S1), and the results were shown in colors. Parameter space that gives “viable” solution is shown in yellow, and parameter spaces that give “inviable” solutions are shown in the other colors. Red color means that the simulation results in M-phase arrest, purple color means G1-arrest, and orange color means abnormal chromosomal separation result, respectively.

We observed that the copy number limit for a mitotic phosphatase gene *CDC14* overexpression was very low (<2), which was well predicted by Chen's model (<2). In contrast, we observed that the copy number limit for the separase gene *ESP1* overexpression was quite high (>160), and the prediction of Chen's model (<1.4) was quite different from the upper limit *in vivo* [13]. According to multiple reports [15]–[18], in Chen's model, enzymes such as Cdc14 phosphatase and separase are regulated by direct 1∶1 binding with their inhibitors (Net1 and Pds1) (Figure 2A and 2C). And overexpression of *CDC14* cured the lethality brought about by overexpression of *NET1* [18]. We thus predict that fragilities upon overexpression of these genes arise from dosage imbalance between the enzymes and their inhibitors [13]. However, it has not been shown that dosage imbalance between *CDC14* and *NET1* causes fragility of the yeast cell cycle. Moreover, there is a discrepancy between predictions of the model and the experimental data in case of the copy number limit of *ESP1* as mentioned above. In this study, we analyzed the molecular mechanisms underlying fragility of *CDC14* regulation and robustness of *ESP1* regulation.

On the basis of our observations, we suggest that dosage imbalance between enzyme and its inhibitor causes cellular fragility. We further suggest that knowledge about cellular robustness can be effectively used to improve integrative mathematical models.

## Results

### Dosage imbalance causes extreme fragility of the yeast cell cycle upon overexpression of *CDC14*

To experimentally determine whether dosage imbalance causes fragility of the yeast cell cycle upon overexpression of certain genes, we first thoroughly analyzed the quantitative relationship between *CDC14* and *NET1*, as well as *ESP1* and *PDS1* using Chen's model. When the regulated enzyme (i.e., Cdc14 or Esp1) alone is overexpressed and its amount exceeds that of its inhibitor (i.e., Net1 or Pds1), the cell cycle halts due to abnormal chromosome separation (Figure S2A and S2C). When the amount of enzyme and its inhibitor increased simultaneously, the cell cycle proceeds normally (Figure S2B and S2D). We performed two parameter viability tests in which parameters for expression of enzymes (Cdc14 and Esp1) and their inhibitors (Net1 and Pds1) were systematically increased and the ability of the cell cycle to persist with any combination of these parameters was tested. Computational analysis showed that to maintain the cell cycle, the absolute amount of enzymes and their inhibitors did not matter, but the quantitative ratio was important and needs to be conserved [(fold increase in expression of *NET1*)/(fold increase in expression of *CDC14*) = 0.95–1.95; (fold increase in expression of *PDS1*)/(fold increase in expression of *ESP1*) = 0.56–2.20] (Figure 2B and 2D). If fragility upon overexpression of *CDC14* is caused by dosage imbalance against *NET1*, this conserved ratio should be observed *in vivo* as well.

**Figure 3:**
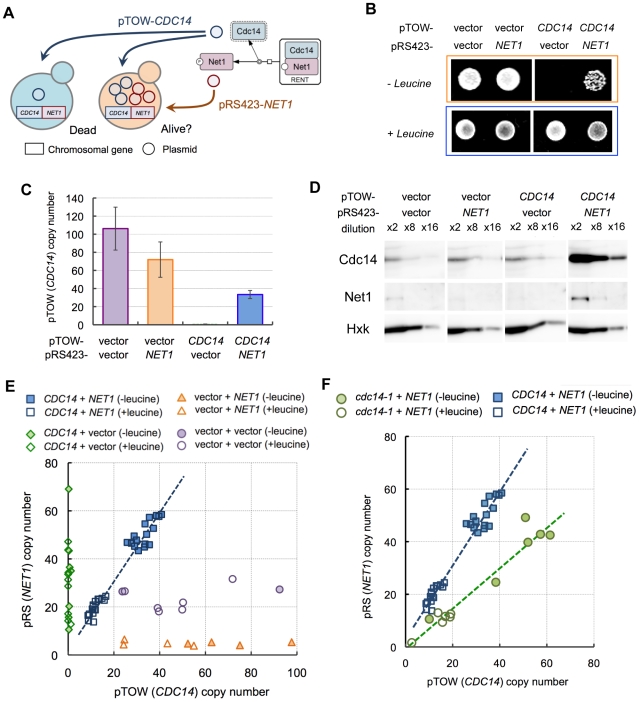
Analysis of dosage relationship between *CDC14* and *NET1* using two-dimensional genetic tug-of-war (2D-gTOW). (A) Schematic representation of 2D-gTOW experiment. (B) Multicopy *NET1* rescued growth defect upon high copy number of *CDC14*. Cells with indicated plasmids were spotted on the SC plate with indicated leucine condition. (C) pTOW (*CDC14*) copy number in 2D-gTOW experiment between *CDC14* and *NET1*. Samples were taken from cells grown under leucine− condition. (D) Quantification of Cdc14 and Net1 proteins from cells grown under conditions as described in C, using western blot analysis. (E) Scatter plot of plasmid copy numbers possessing *CDC14* and *NET1* versus corresponding plasmid vectors in 2D-gTOW experiments. Each plot represents individual experimental trials. (F) Scatter plot of plasmid copy numbers possessing *cdc14-1* and *NET1*.

Similar to computational analysis, we designed an experiment by adding another multicopy plasmid carrying *NET1* into the gTOW experiment of *CDC14* (Figure 3A). This experiment called “2D-gTOW” is based on the fact that multicopy plasmids with *CDC14* or *NET1* replicate with 2µDNA origin, exist as multi-copy in a cell and their numbers vary among the cellular population [19]. Moreover, the copy number of the gTOW plasmid can be controlled by changing leucine concentration in growth media; the average plasmid copy number within a cell is around 35 in the presence of leucine but increases to more than 150 in the absence of leucine (Figure 1) [13].

As expected, introduction of *NET1* plasmid prevented cellular death upon overexpression of *CDC14* (Figure 3B). In the rescued cells, the average plasmid copy number of *CDC14* increased dramatically (∼40 copies per cell; Figure 3C), and the amount of Cdc14 protein also increased accordingly (Figure 3D). We then performed the two parameter viability test by measuring the copy numbers of the plasmids in multiple independent experiments with and without leucine in medium. Most importantly, the ratio between *CDC14* and *NET1* was clearly conserved [(*NET1* copy number)/(*CDC14* copy number) = 1.38, R^2^ = 0.96]] (Figure 3E), similar to that observed in computational analysis. Moreover, when we use *cdc14-1*, a temperature-sensitive *CDC14* gene with reduced activity, the ratio was reduced but still conserved [(*NET1* copy number)/(*cdc14-1* copy number) = 0.77, R^2^ = 0.94] (Figure 3F). We show for the first time that cellular fragility upon overexpression of *CDC14* is caused by dosage imbalance between *NET1* and *CDC14*.

### Cellular fragility upon overexpression of *ESP1* is masked by *CDH1* and *CLB2*

**Figure 4:**
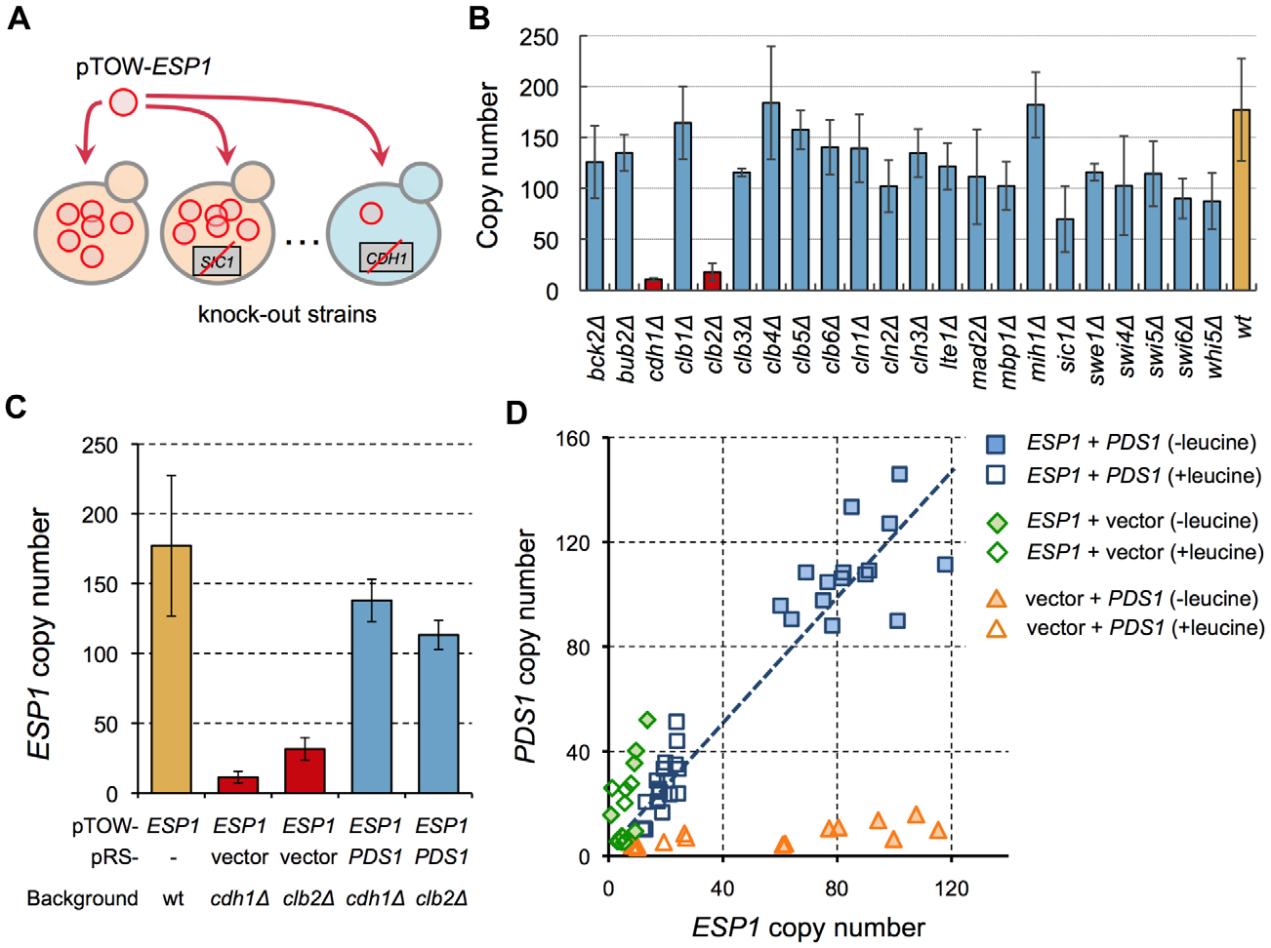
Screening of regulators conferring cellular robustness upon overexpression of *ESP1*. (A) Schematic representation of screening of additional regulators of *ESP1*. (B) Upper limit of copy numbers of *ESP1* in knockout strains of cell cycle-related genes. The pTOW-*ESP1* was introduced into the knockout strains and the plasmid copy number was measured in cells grown under leucine− condition. (C) pTOW-*ESP1* copy numbers in 2D-gTOW experiment between *ESP1* and *PDS1 in cdh1Δ and clb2Δ* mutant cells. Samples were taken from cells grown under leucine− condition. (D) Scatter plot of plasmid copy numbers possessing *ESP1* and *PDS1* in 2D-gTOW experiments. Each plot represents individual experimental trials.

While there is evidence that Esp1 is regulated by 1∶1 binding of Pds1 [15], [16], cellular fragility from dosage imbalance has not been observed (i.e., the copy number limit of *ESP1* overexpression is high). We thus hypothesized that there was an additional regulatory mechanism besides simple binding of Pds1 [13]. To demonstrate *ESP1* regulation by other factors, we performed another 2D-gTOW experiment in various gene knockout mutants (Figure 4A). Among 23 nonessential cell cycle gene knockouts, *cdh1Δ* and *clb2Δ* strain showed significant reduction in the copy number limit of *ESP1* (Figure 4B). The fragility of these knockouts upon overexpression of *ESP1* was rescued by additional *PDS1* plasmids (Figure 4C). Moreover, the ratio between *ESP1* and *PDS1* copy number was well conserved in *cdh1Δ* cells [(*PDS1* copy number)/(*ESP1* copy number) = 1.27, R^2^ = 0.91] (Figure 4D), as observed between *CDC14* and *NET1*. This result indicates that dosage imbalance between *ESP1* and *PDS1* actually causes cellular fragility upon overexpression of *ESP1*, but additional regulations by *CDH1* and *CLB2* mask the potential fragility.

### Computational prediction of *CLB2* function conferring cellular robustness against overexpression of *ESP1*

Our study above indicated the existence of some factors regulating *ESP1* that are not incorporated into Chen's model. We thus tried to improve Chen's model by seeking additional regulations to reproduce the gTOW results. We should note that recently, a study published more detailed model for M-phase-specific regulation [20]. Although this model implements additional regulations such as signaling activity of Esp1 toward FEAR (Cdc14 early anaphase release) pathway (see below), the model still predicted fragility upon overexpression of *ESP1* (Figure S3), indicating the regulation we are seeking is not implemented in this model. We focused on regulation by Clb2, a B-type cyclin-dependent kinase (B-CDK) subunit, because there is evidence that B-CDK is involved in *ESP1* regulation. In the budding yeast, B-CDK phosphorylates the inhibitor Pds1 to regulate its localization [15] and stability in metaphase [21]. In higher eukaryotes, CDK phosphorylates separase (Esp1 homolog) to inhibit its protease activity [22]. However, it has never been shown whether any of these regulations confer cellular robustness upon overexpression of Esp1.

**Figure 5:**
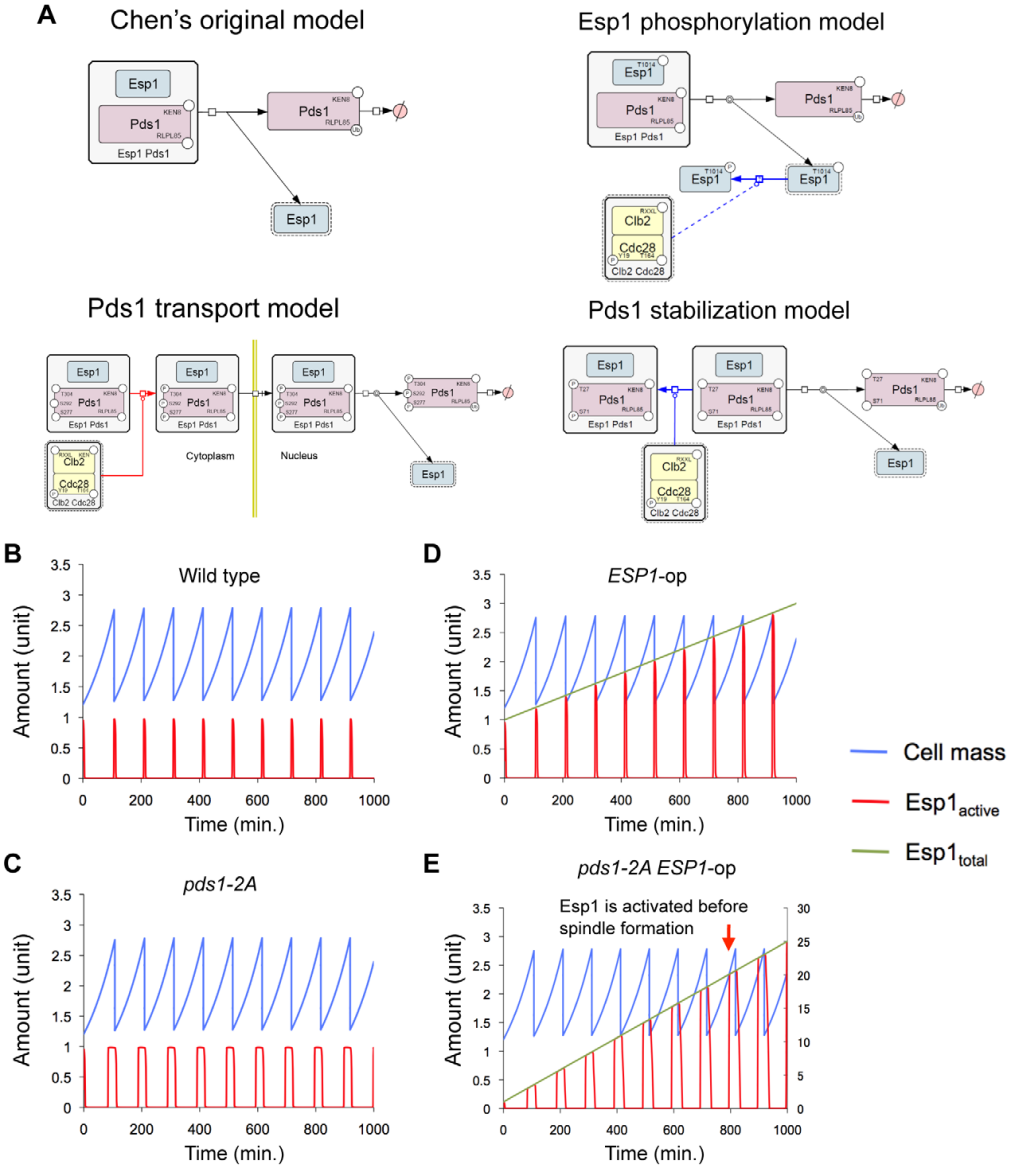
Modified Chen's models and simulation results of Pds1 stabilization model. (A) Graphical notations describing regulation of Esp1 by Pds1 and other factors that were incorporated into Chen's model. The molecular interactions were described as in Figure 2. (B) Time course simulation of modified Chen's model and its (C) *pds1-2A* mutant model (kkp1,pds = 0, kkp2,pds = 0). (D) Time course simulation with gradual increase in the expression of *ESP1* in modified Chen's model and (E) its *pds1-2A* mutant model (kkp1,pds = 0, kkp2,pds = 0). The rate of expression of *ESP1* was increased by 12% of its original value per hour in D, and 2.4% per minute in E. Arrowhead indicates the time when the simulation results in cell cycle failure (*ESP1* is activated before spindle formation). Detailed simulation results are shown in Text S1 and Figure S8.

Therefore, we first modified Chen's model by incorporating each regulation into three independent computational models and tested if they gave viable solution with; overexpression of *ESP1*, overexpression of *ESP1* in the absence of Clb2, and simultaneous overexpression of *ESP1* and *PDS1* (Figure 5A and Table 1) (details in Text S1, S2, S3, S4, S5, S6; Table S1, S2, S3; and Figure S1, S4, S5, S6, S7, S8). Among them, the models for Esp1 phosphorylation and Pds1 stabilization could well reproduce the behaviors of the cell in terms of copy number limits of *ESP1* overexpression (Table 1).

**Table 1:** Modified Chen's models and validation in *in silico* simulation and *in vivo* experiment.

Model name	Reference	Accept high *ESP1*? (Simulation)	Accept high *ESP1* in *clb2Δ*? (Simulation)	Accept high *ESP1* with high *PDS1*? (Simulation)	gTOW experiment
Chen's	[9]	NO (<2)	-	YES (>256)	-
Transport	[15]	YES (>256)	NO (lethal)	NO (<20)	-
Esp1 phosphorylation	[37]	YES (>256)	NO (<2)	YES (>256)	NO^1^
Pds1 phosphorylation	[21]	YES (>135)	NO (<20)	YES (>256)	YES^2^

**Figure 6:**
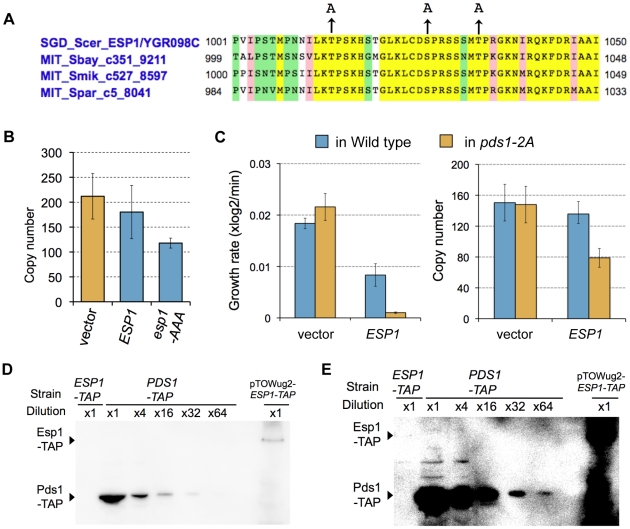
Experimental verification of the predicted models. (A) Conserved putative CDK phosphorylation sites in Esp1 among relative budding yeast species. Alignment was performed using *Saccharomyces* Genome Database, (http://www.yeastgenome.org/). Arrowhead indicates alanine-substituted residues in phosphorylation-negative mutant *esp1-AAA*. (B) Upper limit copy numbers of empty vector, *ESP1*, and *esp1-AAA*, measured in gTOW for cells grown under leucine− condition. (C) Left graph: The growth rate of cells with empty vector and *ESP1* in the wild-type cell (KK001) and cells expressing Pds1-2A (KK002). Right graph: Upper limit copy numbers of empty vector and *ESP1* in the wild-type cell (KK001) and cells expressing Pds1-2A (KK002). (D,E) Quantification of the molecular ratio between Pds1 and Esp1. TAP-tagged each protein expressed from the chromosomal loci were detected by Western blotting. The blot (E) is the same blot as (D), but overexposed to detect 1/64 Pds1-TAP. Because Esp1 expressed from its chromosomal locus could not be detected, *ESP1-TAP* amplified from the chromosomal locus was cloned on to multicopy plasmd (pTOWug2-Esp1-TAP) to confirm the gene was exactly expressing. Judging from the dilution series of Pds1-TAP, Pds1-TAP is at least 64 fold abundant than Esp1-TAP.

**1** Upper limit of copy number of *esp1-AAA* (unphosphorylated form) is high (Figure 6A).

**2** The cell with mutations in Pds1 phosphorylation sites does not accept high copy numbers of *ESP1* (Figure 6C).

### Quantitative regulation of Pds1 through phosphorylation by B-CDK masks fragility arising from dosage imbalance between Esp1 and Pds1

We next experimentally verified these regulations limiting the *ESP1* copy number. When phosphorylation by Clb2 is involved in cellular robustness upon overexpression of *ESP1*, regulation can be destroyed by introducing mutations in phosphorylation sites of the target proteins. In Esp1 of the budding yeast, putative CDK phosphorylation sites [(Thr/Ser)-Pro] are observed, which are conserved among relative yeast species (Thr-1012, Ser-1025, and Thr-1032) (Figure 6A). We substituted these amino acids with alanine (*esp1-AAA*), and measured the copy number limit by gTOW to verify the *ESP1* phosphorylation model, and found that the limit was >100 (Figure 6B). This indicates that direct phosphorylation of Esp1 by Clb2 does not confer robustness upon overexpression of *ESP1*.

**Figure 7:**
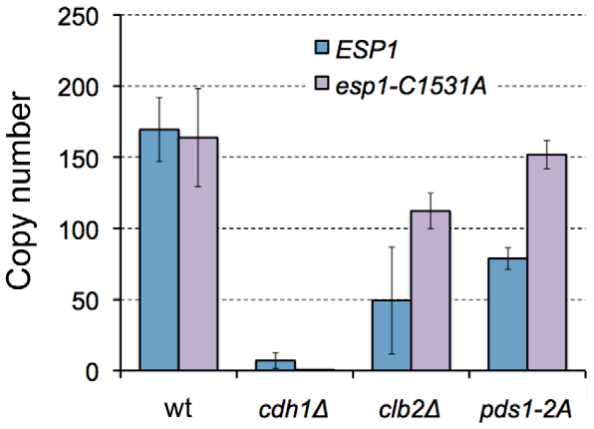
Limits of overexpression of *esp1-C1531A* in wild-type, *clb2Δ*, and *cdh1Δ* mutant cells. Upper limit copy numbers of *ESP1* and *esp1-C1531A* in wild-type, *cdh1Δ*, *clb2Δ*, and *pds1-2A* cells were measured in leucine− condition. Average copy number and SD are large in *clb2Δ* cells in this experiment probably due to reversion mutation.

A study reported that phosphorylation of Pds1 on Thr-27 and Ser-71 by B-CDK stabilizes Pds1, and its regulation is required for synchronization of chromosomal partition [21] (Figure 5A, Pds1 stabilization model). We thus built our model according to their findings, and our model predicted that the copy number limit of *ESP1* was significantly reduced when phosphorylation of Pds1 was inhibited (Figure 5D). We then measured the limit of *ESP1* in the alanine-substituted mutants on these phosphorylation sites (*pds1-2A*), and found that the cell did not accept the high copy number of *ESP1* as observed in *clb2Δ* cells and the limit of overexpression was significantly decreased (Figure 6C). This is the first evidence to show that Pds1 phosphorylation is involved in cellular robustness upon overexpression of *ESP1*. We should note that the decrease of the limit of *ESP1* overexpression in *pds1-2A* cells was not dramatic as in *clb2Δ* cell (Figure 7), suggesting that there is another mechanism by which *clb2* confers cellular robustness against *ESP1* overexpression.

One important but not reported assumption to build the Pds1 stabilization model was that the amount of Pds1 is in large excess of Esp1 (Pds1∶Esp1 is 112∶1 on average during the cell cycle, see Text S1), while their amount is almost the same in Chen's model (0.998∶1). To confirm our assumption, we measured the quantitative ratio of Pds1∶Esp1 using TAP-tagged proteins. Although we could not detect Esp1 expressed from the chromosomal copy, the amount of Pds1 was at leaset more than 64-fold higher than Esp1 (Figure 6D and 6E), supporting our assumption. We thus conclude that quantitative regulation of Pds1 through phosphorylation by B-CDK requires for masking the fragility arising from dosage imbalance between Esp1 and Pds1.

### *CDH1* confers cellular robustness upon overexpression of *ESP1* through the different mechanism from *CLB2*

Esp1 is known to have two independent activities. One is a protease activity to digest certain substrates such as Scc1 and Slk19 [23], [24], and the other is a signaling function to activate FEAR pathway that is a pathway to activate Cdc14 [20], [25]. We thus tested if either of these activities was the determinant of limit of Esp1 overexpression in *cdh1Δ* and *clb2Δ* strains. We measured the limit of *esp1*-*C1531A*, an *ESP1* allele without separase activity [26] in the wild type, *cdh1Δ*, *clb2Δ*, and *pds1-2A* strains respectively. The limit of *esp1*-*C1531A* overexpression was increased in the *clb2Δ* strain and the *pds1-2A* strain up to >100 copies (Figure 7). Interestingly, however, the limit of *esp1*-*C1531A* overexpression was still very low in the *cdh1Δ* strain (Figure 7). These results indicate that *CLB2* and *CDH1* are involved in the robustness of *ESP1* regulation in different ways; *CLB2* is involved in the regulation associated with the protease activity, and *CDH1* is involved in the regulation associated with the FEAR signaling activity.

## Discussion

Knowing the mechanisms causing cellular fragility is important for controlling cellular functions or finding novel drug targets [27], [28]. In this study, we demonstrated that dosage imbalance between Cdc14 and Net1 causes significant cellular fragility upon overexpression of *CDC14* using computational and experimental analysis. We believe that 2D-gTOW can be used as an experimental technique to detect cellular fragility arising from dosage imbalance. As in one of the examples, we were able to detect potential fragility arising from dosage imbalance between *ESP1* and *PDS1*, although it was masked by *CDH1* and *CLB2*. Using this method, we would be able to show more examples of dosage imbalances causing cellular fragility.

Because the strain having mutations on the phosphorylation sites of Pds1 by Clb2 did not accept Esp1 overexpression (Figure 6C), we concluded that the masking function of Clb2 is performed through the stabilization of Pds1. On the other hand, currently we could not explain the masking function of *CDH1*. The function of *CDH1* in Esp1 regulation is at least different from *CLB2*, because the limit of overexpression of the *esp1*-*C1531A* mutant was still low in the *cdh1Δ* strain, but high in the *clb2Δ* strain (Figure 7). This fact suggests that the masking function of *CDH1* is performed through the process downstream of FEAR pathway, which regulates the activity of Cdc14 phosphatase [25]. Cdh1 is a component of ubiquitin-conjugating enzyme complex called APC that degrades a number of proteins such as Clb2, Cdc5, Cdc20, Cin8, etc. [29]–[33]. *CDH1* will thus confer the robustness of Esp1 regulation through degradation of these M-phase components.

One possible function of Cdh1 to confer cellular robustness against the overexpression of Esp1 is performed thorough a polo-like kinase Cdc5, which also regulates Cdc14 activity [34]. When Cdh1 is inactivated, the substrate Cdc5 activity will increase, and Cdc14 will be activated. In the situation, the cell will be very sensitive against further activation of Cdc14 by the FEAR pathway due to the overexpression of Esp1. Alternatively, the masking function of Cdh1 could be performed through Cdc20. Cdc20 is another component of APC, which promotes the degradation of Pds1 [35]. Because Cdc20 is a potential target of Cdh1 [32], [33], the activity of Cdc20 could be higher in the *cdh1Δ* strain. It is thus possible that the amount of Pds1 is reduced in the *cdh1Δ* strain due to the over-activation of Cdc20, which causes reduction of the robustness of *ESP1* regulation.

In addition to the mechanisms described above, there could be other mechanisms that make the cellular system robust against Esp1 overexpression. For example, Pds1 is considered as a chaperone for Esp1 [36], [37], which will make excess Esp1 over Pds1 unstable. Although we did not adopt the Pds1 transport model (Figure 6) to explain our finding, it is also a quite effective mechanism to regulate the activity of Esp1. *CDC55*, a component of PP2A phosphatase and a direct regulator and a downstream effector of Esp1 [20], [38]), will be also involved in the robustness. M-phase regulations with components such as Cdc5 and Cdc55 should be implemented into the integrated model, and verified further combinational gTOW experiments to uncover the whole regulatory mechanisms conferring the cellular robustness against Esp1 overexpression.

We should note that the reason why the *clb2Δ* cell and *cdh1Δ* cell are fragile against overexpression of *ESP1*, could be arisen from the same mechanistic consequence as the observation that *clb2Δ* and *cdh1Δ* are synthetic lethal with *pds1Δ* [39], [40], although Pds1 phosphorylation by Clb2 should be an exception. Unfortunately, Chen's model and our modified model do not reproduce the behavior of *pds1Δ* mutant (i.e., viable in real cell, but essential in the models [9], data not shown). We thus could not test these phenotypes in our model. We hope that modifications of the model by implementing the regulation above will solve the discrepancy.

In the budding yeast cell, there are several genes, such as actin encoding gene (*ACT1*) or beta-tubulin-encoding gene (*TUB2*), that cause extreme fragility due to imbalance against binding partners [41], [42]. Dosage balance (i.e., stoichiometry) between histone dimmer sets must be conserved for normal mitotic chromosome transmission [43]. We thus hypothesize that dosage imbalance is a common cause of cellular fragility. In regulation of *CDC14*, dosage imbalance is exposed whereas in regulation of *ESP1*, it is masked. In many cellular processes, it is likely that fragilities caused by regulation through 1∶1 binding (here we call “stoichiometric regulation”) will be masked. In the case of Esp1, what we found here (and Chen's model did not implement) was that the inhibitor Pds1 was in large excess of the separase Esp1 (Figure 6). Excess of the inhibitor could be a general mechanism by which the systems are robust against dosage fluctuation of the enzyme. In case of Cdc14 and Net1, the amount of both proteins within the cell are the same order (Net1-TAP exists with 1.59E+03 molecules/cell and Ccd14-TAP exists with 8.55E+03 molecules/cell) [44], this is one of the reasons of the exposed fragility. However, as a trade off, the excess inhibitor should be effectively and timely inactivated when activation of the enzyme is required. Separase needs to be activated accurately in the period of metaphase to anaphase transition. Phosphorylation of Pds1 on Thr-27 and Ser-71 by Clb2 is the regulation that makes the precise inactivation (degradation) of Pds1, which requires the cell cycle system to be robust against overexpression of Esp1. Regulations conferring cellular robustness might therefore be generally discovered around stoichiometric regulations, as was observed in case of *ESP1*. Moreover, we may be able to control cellular robustness by modifying the regulators around stoichiometric regulation.

How is fragile regulation advantageous for a cell? Regulation by simple protein-protein interactions is one of the simplest ways to generate ultrasensitive responses in cellular systems [45], [46], and might have evolved to add novel regulations toward enzymes. For example, multiple CDK inhibitors are present in yeasts to mammalian cells, but they are quite diverse. While B-type cyclins Clb2 (*S. cerevisiae*) and Cdc13 (*S. pombe*) are quite similar (BLAST E-value 6e-79), their inhibitors Sic1 and Rum1 do not show any similarity (BLAST E-value >0.05). This suggests that these factors have evolved independently from different ancestor proteins to achieve the common purpose of binding and inhibiting CDK. In addition, drugs for molecular targeted therapy utilize the mechanism of stoichiometric regulation against the target. This is the only known enzymatic regulation thus far that humans can design. In fission yeast and higher eukaryotes, no stoichiometric regulator for Cdc14 phosphatase homologue is known to exist [47]. We propose that during evolution, the budding yeast uniquely acquired Cdc14 regulation with Net1, but it conversely produced fragility caused by dosage imbalance as a trade-off. The activity of Cdc14 itself is quite tightly regulated by two signalling pathway designated FEAR and MEN (mitotic exit network), which are found only in the budding yeast [48]. The budding yeast may have uniquely acquired these regulations in order to buffer the fragility due to the dosage imbalance.

Developing integrative cellular models with high predictive ability is one of the goals of systems biology. However, it is sometimes criticized that large-scale integrative cellular models are indefinitely adjustable and can no longer be proven false [49]. For this purpose, a general experimental technique to effectively evaluate and refine models is needed. In this study, we evaluated a model with data for cellular robustness obtained by gTOW, found discrepancies, modified them according to the current knowledge for reproducing robustness, and evaluated them with combinatorial gTOW. We believe that this analytical scheme will be effective for further development of integrative cellular models.

## Materials and Methods

### Yeast strains and growth conditions

A wild-type yeast strain BY4741 (*MAT*a, *his3Δ1*, *leu2Δ0*, *met15Δ0*, *ura3Δ0*) and its derivatives with deletion of cell-cycle-related genes (in Figure 4) were obtained from Open Biosystems Inc. Haploid yeast strains KK001 (*leu2Δ*, *ura3Δ*, *PDS1*) and KK002 (*leu2Δ*, *ura3Δ*, *pds1-T27A*, *S71A*) are progenies of LH651 and LH557 [21], respectively. To detect TAP-tagged Pds1 and Esp1, derivatives of a yeast strain SC0000 (*MAT*a, *ade2*, *arg4*, l*eu2-3,112*, *trp1-289*, *ura3-52*), SC4998 (*PDS1-TAP*-*klURA3*) and SC1033 (*ESP1-TAP*-*klURA3*)(Euroscarf) were used. Yeast cells were cultured in synthetic complete medium (SC) lacking indicated amino acids. SC medium was prepared using YNB with ammonium sulfate (MP Biomedicals, LLC) with DO supplement (Clontech) and 2% glucose.

### Plasmid constructions

Plasmids used in this study are listed in Table 2. pTOWug2 is a pSBI40 derivative carrying *URA3-GFP* fusion gene instead of *URA3*. pRS423-mRFP is a pRS423 derivative carrying *HIS3-RFP* fusion gene instead of *HIS3*.

**Table 2:** Plasmids used in this study.*1

Plasmid Name	Gene	Up primer*2	Down primer*2	Description and Reference
pSBI40				[13]
pTOWug2				pSBI40 *URA3-GFP*
pRS423				[52]
pRS423mRFP				pRS423 *HIS3-RFP*
pTOW-*CDC14*	*CDC14*			[13]
pTOW-*cdc14-1*	*cdc14-1*	OSBI0505	OSBI0045	[53]
pTOWug2-*ESP1*	*ESP1*	OSBI0083	OSBI0084	Present study
pTOWug-esp1-C1531A	esp1-C1531A	OSBI0083OSBI0561	OSBI0562OSBI0084	Present study
pTOWug2-*esp1-AAA*	esp1-3A	OSBI0083OSBI0919	OSBI0920OSBI0084	T1013A, S1026A, and T1022A. Present study
pTOWug2-*ESP1-TAP*	ESP1-TAP	OSBI0083	OSBI0084	Amplified from SC1033 genome.Present study
pRS423-*NET1*	*NET1*	OSBI0156	OSBI0157	Present study
pRS423mRFP-*PDS1*	*PDS1*	OSBI0081	OSBI0082	Present study

*1 All plasmids were constructed using the gap-repair method using primers listed as described previously [13].

*2 The sequence of the primers used are described previously [13], except OSBI0561 (CCCCCAGCTTTTTACTGGGCgcGTCTTCAGCAGCGATGAAAT), OSBI0562 (ATTTCATCGCTGCTGAAGACgcGCCCAGTAAAAAGCTGGGGG), OSBI0919 (CTCCTTCCAAGCATAGTACAGGATTGAAGCTTTGCGATgCACCAAGATCGTCGAGCATGgCGCCTAGAGGTAAGAATATA) and OSBI0920 (CATGCTCGACGATCTTGGTGcATCGCAAAGCTTCAATCCTGTACTATGCTT).

### gTOW procedure

gTOW experiments were performed as described previously [13]. For 2D-gTOW, cells transformed with both pSBI40 and pRS423 derivatives were cultivated in SC without uracil and histidine, and then they were transferred into SC without uracil, histidine, and leucine. The copy numbers of pSBI40 and pRS423 derivatives were measured using real-time PCR as described previously [13], except that an *HIS3* primer set (*HIS3*-1F, TTCCGGCTGGTCGCTAAT and *HIS3*-1R, GCGCAAATCCTGATCCAAAC) was used to measure the copy number of pRS423 derivatives. Data shown in Figure 3C, Figure 4B and 4C, Figure 6B and 6C, and Figure 7 are averages of at least four independent experiments.

### Quantification of proteins

Cdc14 and Net1 proteins were quantified by western blot analysis using their specific antibodies (sc12045 and sc27758; Santa Cruz Biotechnology, Inc.) as described previously [13]. TAP-tagged proteins were detected using PAP (P1901l; Sigma-Aldrich).

### Computation

Numerical simulations were carried out using Matlab version 7.3.0. Chen's model and Queralt's model were implemented on Matlab script files [9], [20]. The code for Chen's model was based on that obtained from Dr. Cross. For details and codes used in this study refer to Text S1.

## Supporting Information

Figure S1 Viability test in Chen's model. Detailed interpretation is described in Text S1 (“Viability test”).
(0.84 MB PDF)

Figure S2 Time course simulation of Chen model with over-expression of its components. (A–D) Time course simulation with gradual increase of the expression of *CDC14* alone (A, parameter ks,14), both *CDC14* and *NET1* (B, parameter k_s,14_ and k_s,net_), *ESP1* alone (C, parameter [Esp1]_T_), and both *ESP1* and *PDS1* (D, parameter [Esp1]_T_, k_′s,pds_, k_″s1,pds_, and k_″s2,pds_). Each parameter was increased at the rate of 12% of its original value per hour. Arrowhead indicates the timing when the simulation results in the cell cycle failure (abnormal chromosomal segregation at time).
(1.02 MB PDF)

Figure S3 Prediction of the behavior of *Esp1* regulatory module in Queralt's model. (A) Two parameter viability test of Queralt's model. Simulation results are shown in colors when the expression levels (copy numbers) of *ESP1* and *PDS1* increased. The x-axis is the fold increase in transcription of *ESP1*(k_s,separase_) and the y-axis is that in transcription rates of *PDS1* (k_s,separase_). Cell was considered to be viableonly if both sister chromosome segregation (the concentration of *Esp1* to increase above 0.1) and nuclear division (the concentration of Clb2 dropping below 0.3) were executed in this order. (B,C) Time course simulation of Queralt's model when *ESP1* (B) or both *ESP1* and *PDS1* (C) is/are over-expressed (5 fold).
(0.71 MB PDF)

Figure S4 Chen's original model and its time course simulation. (A) Process diagram describing the regulation of Esp1 by Pds1 and other factors. The diagram was drawn using CellDesigner4.0. (B) Time course simulation of wild type strain. (C,D) Time course simulation with gradual increase of *ESP1* expression alone (C), and both *ESP1* and *PDS1* (D) at the rate of 12% of its original value per hour.
(0.92 MB PDF)

Figure S5 Pds1 transport model and its time course simulation. (A) Process diagram describing the regulation of Esp1 by Pds1 and other factors. The diagram was drawn using CellDesigner4.0. (B) Time course simulation of wild type strain. (C,D) Time course simulation with gradual increase of *ESP1* expression alone (C), and both *ESP1* and *PDS1* (D) at the rate of 5% per minute.
(1.07 MB PDF)

Figure S6 Esp1 phosphorylation model and its time course simulation. (A) Process diagram describing the regulation of Esp1 by Pds1 and other factors. The diagram was drawn using CellDesigner4.0. (B) Time course simulation of wild type strain. (C) Time course simulation with gradual increase of *ESP1* expression alone at the rate of 12% of its original value per hour.
(0.97 MB PDF)

Figure S7 Pds1 stabilization model and its time course simulation. (A) Process diagram describing the regulation of Esp1 by Pds1 and other factors. The diagram was drawn using CellDesigner4.0. (B) Time course simulation of wild type strain. (C) Time course simulation with gradual increase of *ESP1* expression alone at the rate of 12% of its original value per hour.
(0.88 MB PDF)

Figure S8 Detailed simulation result of the Pds1 stabilization model. (A) wild type model (viable), (B) wild type model with *ESP1*×100 (viable), (C) *pds1-2A* model (viable), and (D) *pds1-2A* model with *ESP1*×30 (enviable due to the ordering error: abnormal chromosome separation). Each event is numbered as; (3)Spindle alignment ([SPN] increase through 1), (4) Sister chromosome separation ([Esp1] increases through 0.1), and (5) Cell division ([Clb2] decreases through 0.3).
(1.70 MB PDF)

Table S1 Parameter set (1/3) for simulating the “stabilization model”.
(0.02 MB XLS)

Table S2 Parameter set (2/3) for simulating the “stabilization model”.
(0.02 MB XLS)

Table S3 Parameter set (3/3) for simulating the “stabilization model”.
(0.03 MB XLS)

Text S1 Supplementary methods for computation; viability test; examination of the possible *ESP1* regulation by *CLB2*.
(0.10 MB DOC)

Text S2 Matlab m-file (1/5) for simulating the “stabilization model”.
(0.01 MB TXT)

Text S3 Matlab m-file (2/5) for simulating the “stabilization model”.
(0.01 MB TXT)

Text S4 Matlab m-file (3/5) for simulating the “stabilization model”.
(0.02 MB TXT)

Text S5 Matlab m-file (4/5) for simulating the “stabilization model”.
(0.01 MB TXT)

Text S6 Matlab m-file (5/5) for simulating the “stabilization model”.
(0.01 MB TXT)
