# Supplementary material for: Fragilities Caused by Dosage Imbalance in Regulation of the Budding Yeast Cell Cycle
Source: PLoS Genet. 2010 Apr 22;6(4):e1000919. doi: 10.1371/journal.pgen.1000919 (PMC2858678; doi:10.1371/journal.pgen.1000919)
Supplement: Figure S1 — Viability test in Chen's model. Detailed interpretation is described in Text S1 (“Viability test”). (0.84 MB PDF) [file pgen.1000919.s001.pdf]

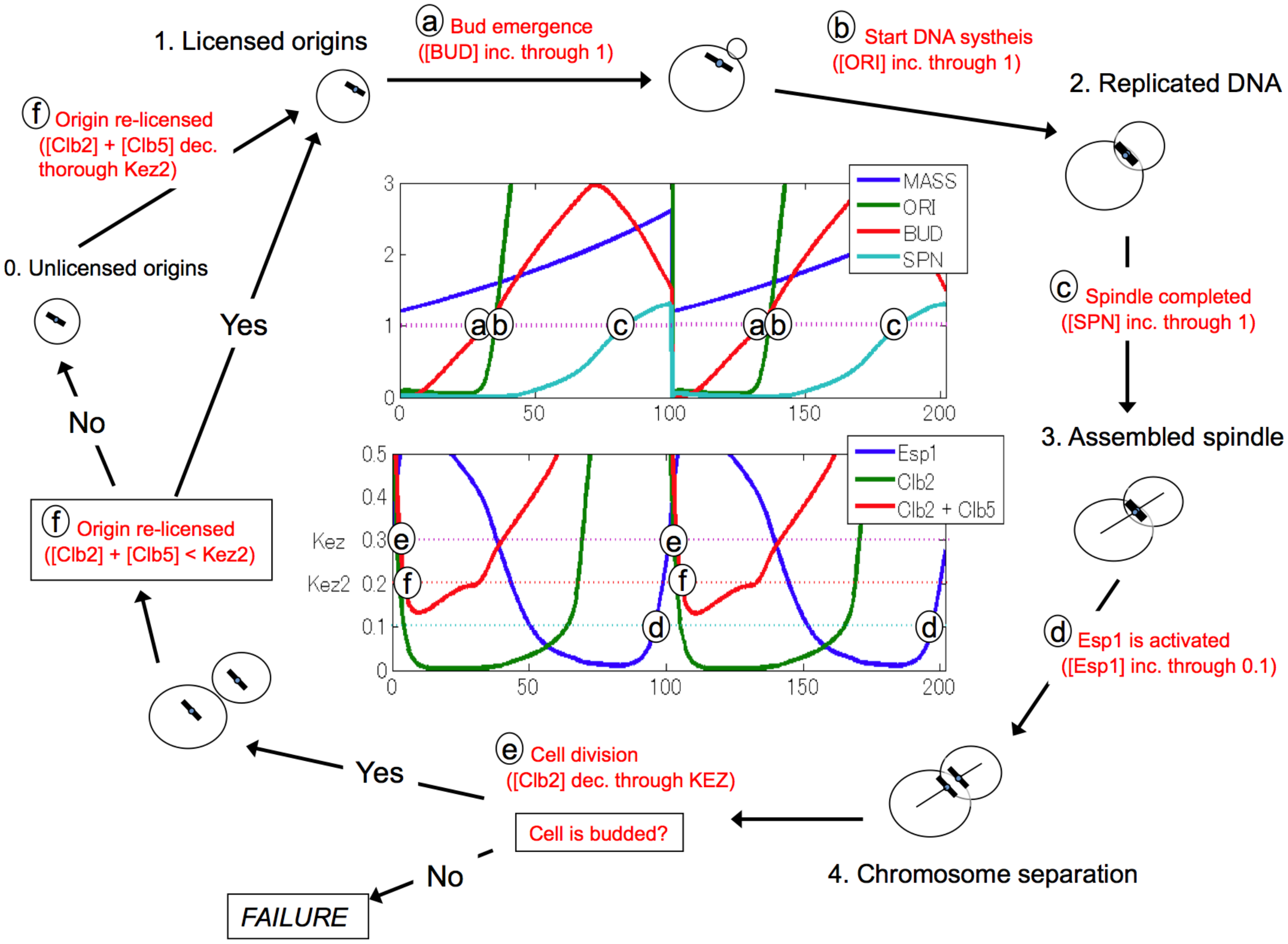

Figure S1. Viability test in Chen's model. Detailed interpretation is described in Supplementary Notes ("Viability test")
