# Supplementary material for: Fragilities Caused by Dosage Imbalance in Regulation of the Budding Yeast Cell Cycle
Source: PLoS Genet. 2010 Apr 22;6(4):e1000919. doi: 10.1371/journal.pgen.1000919 (PMC2858678; doi:10.1371/journal.pgen.1000919)
Supplement: Figure S2 — Time course simulation of Chen model with over-expression of its components. (A–D) Time course simulation with gradual increase of the expression of CDC14 alone (A, parameter ks,14), both CDC14 and NET1 (B, parameter ks,14 and ks,net), ESP1 alone (C, parameter [Esp1]T), and both ESP1 and PDS1 (D, parameter [Esp1]T, k′s,pds, k″s1,pds, and k″s2,pds). Each parameter was increased at the rate of 12% of its original value per hour. Arrowhead indicates the timing when the simulation results in the cell cycle failure (abnormal chromosomal segregation at time). (1.02 MB PDF) [file pgen.1000919.s002.pdf]

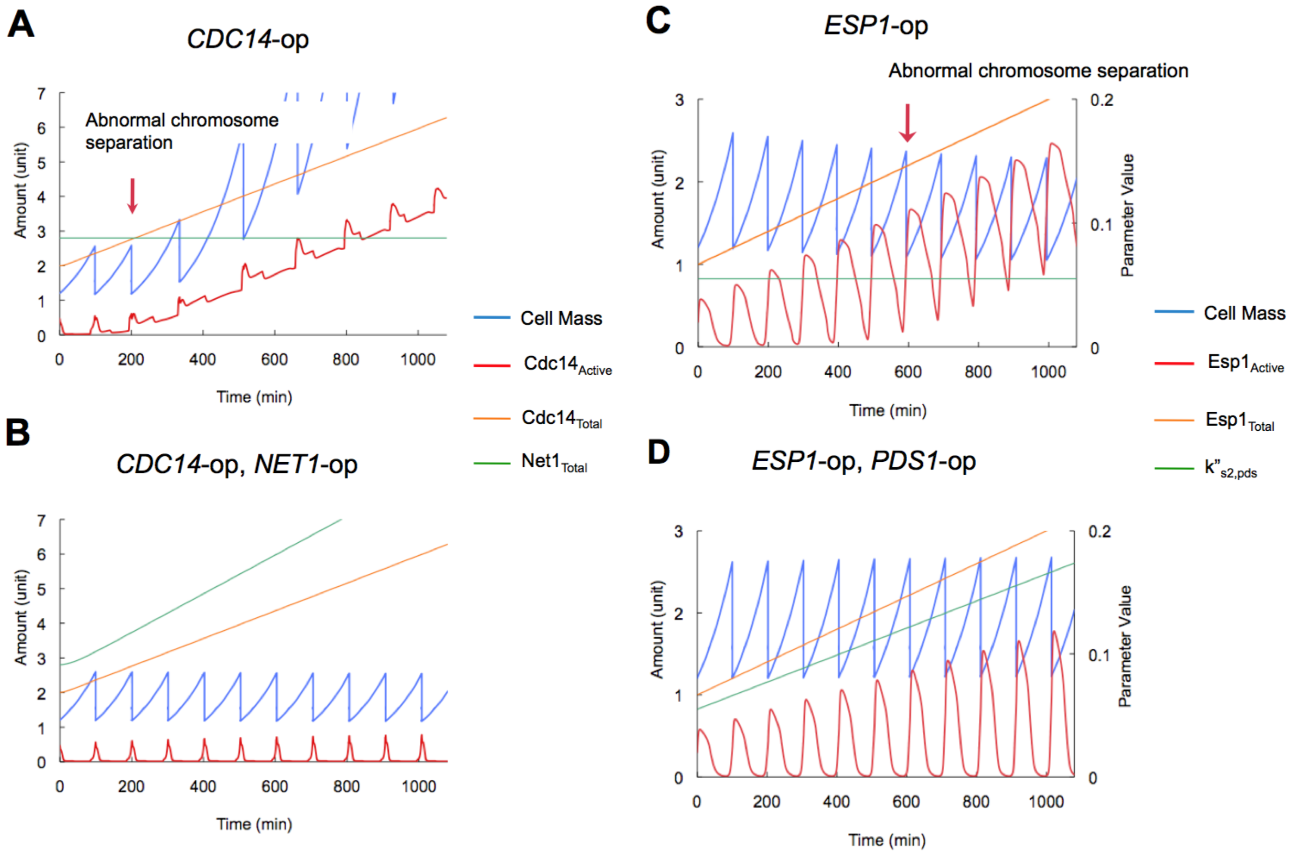

Figure S2. Time course simulation of Chen's model with over-expression of its components. (A-D) Time course simulation with gradual increase of the expression of *CDC14* alone (**A**, parameter $k_{s,14}$), both *CDC14* and *NET1* (**B**, parameter $k_{s,14}$ and $k_{s,net}$), *ESP1* alone (**C**, parameter $[Esp1]_T$), and both *ESP1* and *PDS1* (**D**, parameter $[Esp1]_T$, $k'_{s,pds}$, $k''_{s1,pds}$ and $k''_{s2,pds}$). Each parameter was increased at the rate of 12% of its original value per hour. Arrowhead indicates the timing when the simulation results in the cell cycle failure (abnormal chromosomal segregation at time).
