# Supplementary material for: Fragilities Caused by Dosage Imbalance in Regulation of the Budding Yeast Cell Cycle
Source: PLoS Genet. 2010 Apr 22;6(4):e1000919. doi: 10.1371/journal.pgen.1000919 (PMC2858678; doi:10.1371/journal.pgen.1000919)
Supplement: Figure S3 — Prediction of the behavior of Esp1 regulatory module in Queralt's model. (A) Two parameter viability test of Queralt's model. Simulation results are shown in colors when the expression levels (copy numbers) of ESP1 and PDS1 increased. The x-axis is the fold increase in transcription of ESP1(ks,separase) and the y-axis is that in transcription rates of PDS1 (ks,separase). Cell was considered to be viableonly if both sister chromosome segregation (the concentration of Esp1 to increase above 0.1) and nuclear division (the concentration of Clb2 dropping below 0.3) were executed in this order. (B,C) Time course simulation of Queralt's model when ESP1 (B) or both ESP1 and PDS1 (C) is/are over-expressed (5 fold). (0.71 MB PDF) [file pgen.1000919.s003.pdf]

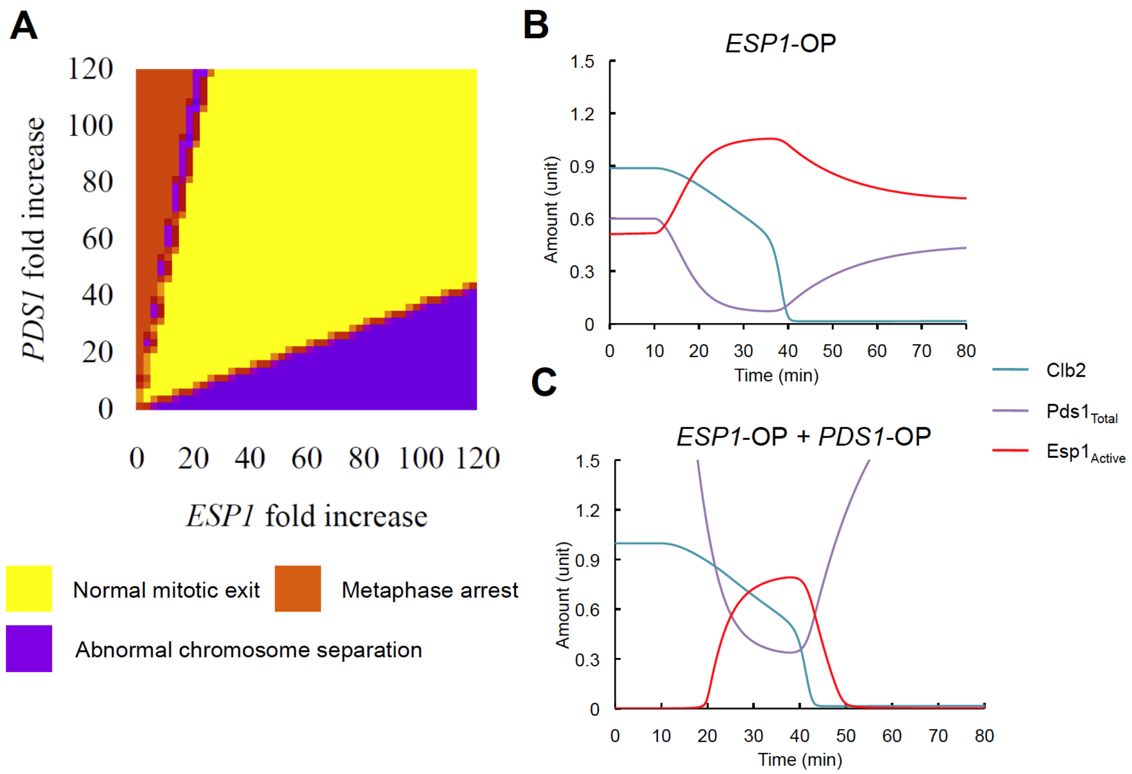

Figure S3. Prediction of the behavior of Esp1 regulatory module in Queralt's model. (**A**) Two parameter viability test of Queralt's model. Simulation results are shown in colors when the expression levels (copy numbers) of *ESP1* and *PDS1* increased. The x-axis is the fold increase in transcription of *ESP1* ($k_{s,separase}$) and the y-axis is that in transcription rates of *PDS1* ($k_{s,separase}$). Cell was considered to be viable only if both sister chromosome segregation (the concentration of Esp1 to increase above 0.1) and nuclear division (the concentration of Clb2 dropping below 0.3) were executed in this order. (**B** and **C**) Time course simulation of Queralt's model when *ESP1* (**B**) or both *ESP1* and *PDS1* (**C**) is/are over-expressed (5 fold).
