# Supplementary material for: Fragilities Caused by Dosage Imbalance in Regulation of the Budding Yeast Cell Cycle
Source: PLoS Genet. 2010 Apr 22;6(4):e1000919. doi: 10.1371/journal.pgen.1000919 (PMC2858678; doi:10.1371/journal.pgen.1000919)
Supplement: Figure S5 — Pds1 transport model and its time course simulation. (A) Process diagram describing the regulation of Esp1 by Pds1 and other factors. The diagram was drawn using CellDesigner4.0. (B) Time course simulation of wild type strain. (C,D) Time course simulation with gradual increase of ESP1 expression alone (C), and both ESP1 and PDS1 (D) at the rate of 5% per minute. (1.07 MB PDF) [file pgen.1000919.s005.pdf]

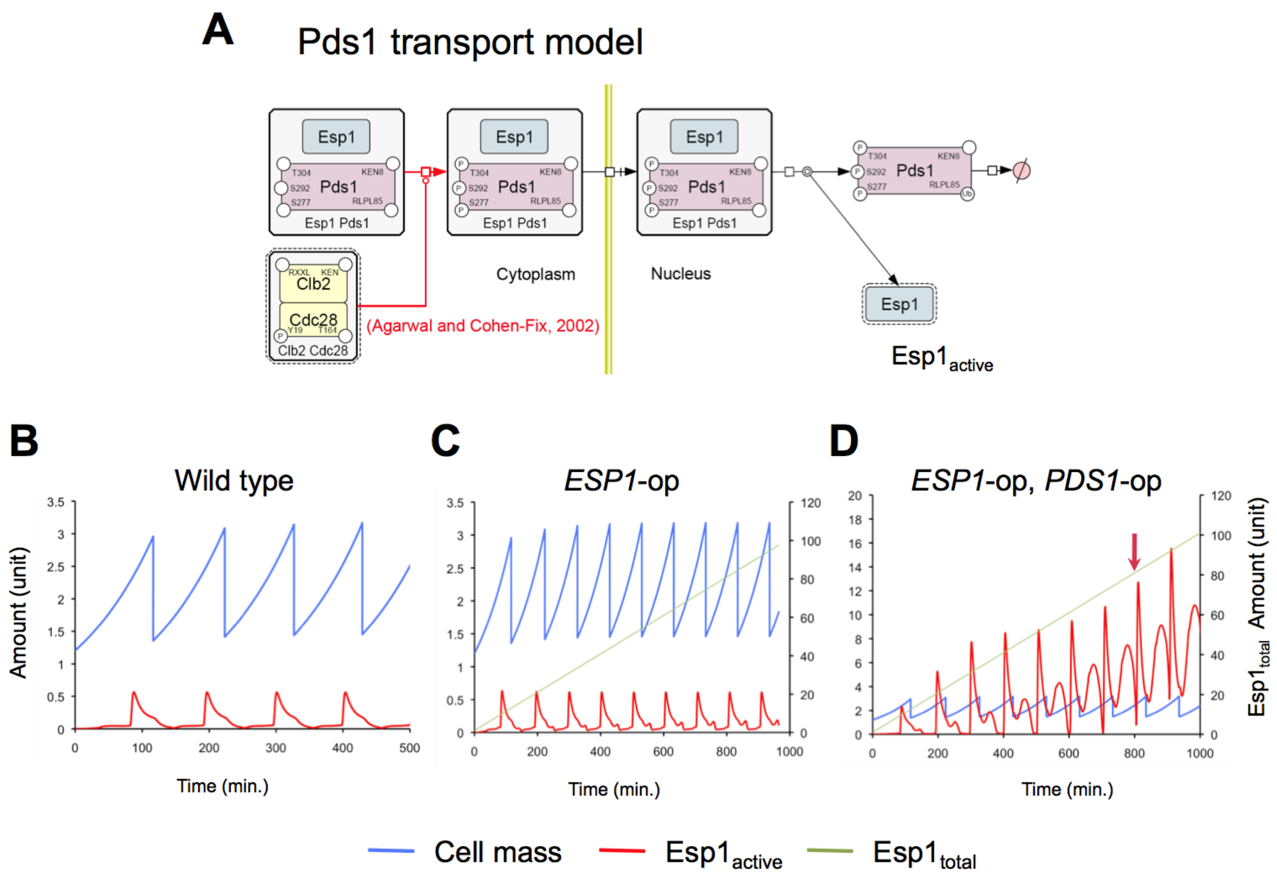

Figure S5. Pds1 transport model and its time course simulation. (**A**) Process diagram describing the regulation of Esp1 by Pds1 and other factors. The diagram was drawn using CellDesigner4.0. (**B**) Time course simulation of wild type strain. (**C**-**D**) Time course simulation with gradual increase of *ESP1* expression alone (**C**), and both *ESP1* and *PDS1* (**D**) at the rate of 5% per minute.
