# Supplementary material for: Fragilities Caused by Dosage Imbalance in Regulation of the Budding Yeast Cell Cycle
Source: PLoS Genet. 2010 Apr 22;6(4):e1000919. doi: 10.1371/journal.pgen.1000919 (PMC2858678; doi:10.1371/journal.pgen.1000919)
Supplement: Figure S8 — Detailed simulation result of the Pds1 stabilization model. (A) wild type model (viable), (B) wild type model with ESP1×100 (viable), (C) pds1-2A model (viable), and (D) pds1-2A model with ESP1×30 (enviable due to the ordering error: abnormal chromosome separation). Each event is numbered as; (3)Spindle alignment ([SPN] increase through 1), (4) Sister chromosome separation ([Esp1] increases through 0.1), and (5) Cell division ([Clb2] decreases through 0.3). (1.70 MB PDF) [file pgen.1000919.s008.pdf]

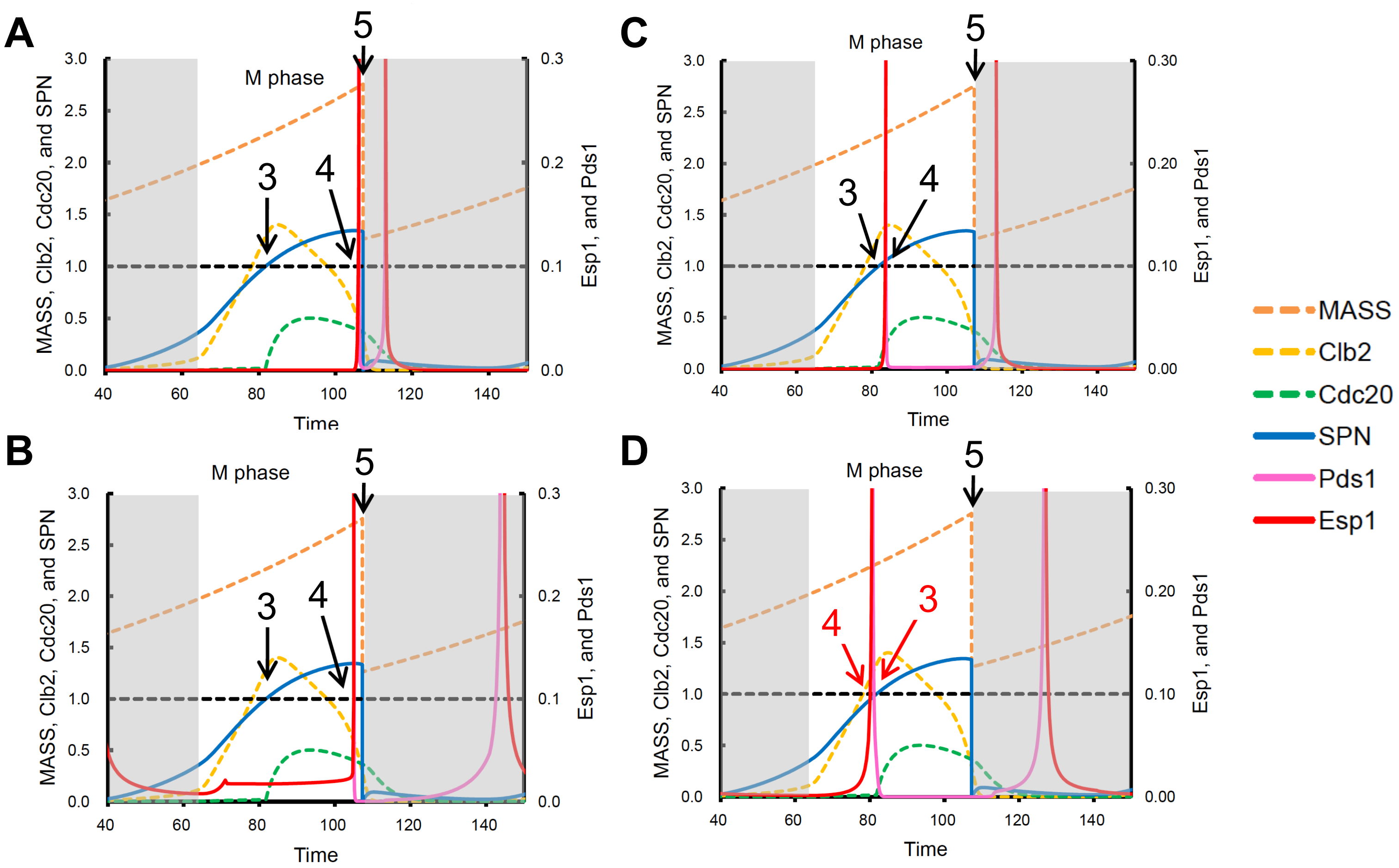

Figure S8. Detailed simulation result of the Pds1 stabilization model. (**A**) wild type model (viable), (**B**) wild type model with *ESP1*x100 (viable), (**C**) *pds1-2A* model (viable), and (**D**) *pds1-2A* model with *ESP1*x30 (enviable due to the ordering error: abnormal chromosome separation). Each event is numbered as; (3) Spindle alignment ([SPN] increase through 1), (4) Sister chromosome separation ([Esp1] increases through 0.1), and (5) Cell division ([Clb2] decreases through 0.3).
