# Supplementary material for: Fragilities Caused by Dosage Imbalance in Regulation of the Budding Yeast Cell Cycle
Source: PLoS Genet. 2010 Apr 22;6(4):e1000919. doi: 10.1371/journal.pgen.1000919 (PMC2858678; doi:10.1371/journal.pgen.1000919)
Supplement: Text S1 — Supplementary methods for computation; viability test; examination of the possible ESP1 regulation by CLB2. (0.10 MB DOC) [file pgen.1000919.s012.doc]

**Supplementary Notes**

**Contents**

1. Supplementary Methods for Computation

2. Viability test

3. Examination of the possible *ESP1* regulation by *CLB2*

3-1. Chen’s model (original model)

3-2. Pds1 transport model

3-3. Esp1 phosphorylation model

3-4. Pds1 stabilization model

4. References

**1. Supplementary Methods for Computation**

Matlab scripts of the refined model used in this study are Text S2~S5 and Table S1~S3. Matlab scripts of other models (Chen’s model [1]), and the model described in Queralt’s et al. [2], and two hypothetical models; (see below) are also available upon request. The codes were tested on Matlab version 7.3.0 with Microsoft Office 2000 (or later) on Windows XP.

Before simulation, save Table S1~S3 as a single Excel file named “model_init.xls” with three worksheets, rename Text S2 setup_model.m, Text S3 model_ode.m, Text S4 singlerun.m, Text S5 test_vaialility.m, Text S6 quantify_limits.m. setup_model.m and model_init.xls are setup files, model_ode.m is a model file, singlerun.m is a script for running a simulation, and test_vaialility.m and quantify_limits.m are scripts for robustness analysis.

A function setup_model reads an Excel file if it exists (model_init.xls) and returns a “Structure” consisting of model parameters. Functions for running a simulation (singlerun, test_viability, quantify_limits) need the model loaded by setup_model as the first argument, and they are initialized with it. A function singlerun carries out one individual simulation, and returns its results (phenotype, period, and volume at the final division). singlerun plots time course data if value of the second argument is “true”. quantify_limits determines the upper limit dosage of parameters indicating expression of 21 genes by bisection method on logarithmic scale (base 2) to the tolerance 0.01, and save them to a file (indicated with the second argument). test_viability carries out 2D viability testing on linear scale over the range of fold increase 0-120 at steps of 2, and save its result to a file (the second argument). These robustness analyses followed the rules of viability described in Chen et al. [1] (see below). Parameters for daughter cell on glucose medium were used for all simulations in this work.

**2. Viability test**

The rules of viability in Chen’s model are followed as described [1]. When the simulation results in the following 4 states, the model is considered as “inviable” (others are “viable”).

(1) Following events do not occur in the order shown below.

1. Replication origin re-licensing ([Clb2] + [Clb5] decreases through Kez2) (ⓕ in Figure S1)
2. Replication origin activation ([Clb2] + [Clb5] increases through 1) (ⓑ in Figure S1)
3. Spindle alignment ([SPN] increases through 1)(ⓒ in Figure S1)
4. Sister chromosome separation ([Esp1] increases through 0.1 due to Pds1 degradation)(ⓓ in Figure S1).
5. Cell division ([Clb2] decrease through < Kez)(ⓔ in Figure S1).

(2) Cell divides without budding (i.e. [BUD] <1, event ⓐ in Figure S1).

(3) The model does not show cyclic solution.

(4) The cell size reaches >10.

We consider the inviable model halts the cell cycle due to; G1-arrest when the cell size reaches >10 before DNA replication, and M-arrest when the cell size reaches >10 after DNA replication, and abnormal chromosome segregation when the above ordering error of 3 → 4 → 5 occurs after DNA replication. Examples of the viability judgment are shown in Figure S8.

**3. Examination of the possible *ESP1* regulation by *CLB2***

*CLB2* encodes a regulatory subunit of the cyclin dependent kinase (CDK). There are experimental reports showing that Esp1 itself and Pds1 could be substrates of B-CDK. We thus modified Chen’s model according to the reports (below), and examined the likelihood of the hypotheses using computer simulation and gTOW experiments. The results were summarized in Table 1.

**3-1. Chen’s model (original model)**

In Chen’s model, as described in the main text, Esp1 is only regulated by the stoichiometric inhibition with Pds1 (Figure S4A), and thus the model do not accept the high copy *ESP1* (upper limit is less than 2 fold increase) (Figure S4C).

**3-2. Pds1 transport model**

It is reported that the CDK dependent phosphorylation of Pds1 on Ser-277, Ser-292, and Thr-304 enhances the binding of Pds1 with Esp1 and the transport of Esp1 and Pds1 into the nucleus [3]. We thus modified Chen’s model to reproduce the Pds1 phosphorylation and the transport of Esp1 and Pds1 into the nucleus.

In the model; 1) Pds1 is phosphorylated to become Pds1P and PEP (a complex of Esp1 and Pds1P), 2) while Pds1 can be transported into the nucleus by itself, Esp1 cannot be transported into the nucleus by itself, 3) Proteins in the nucleus are described as Esp1nuc and Pds1nuc, and the amounts of cyclins, Cdc14, and the anaphase promoting complex (APC) were consistent in the nucleus and cytoplasm, 4) The FEAR activity is constant to simplify the prediction, 5) Because Esp1 is considered to function in the nucleus, Esp1nuc is used as the active form of Esp1 (Figure S5A).

Changes made from Chen’s model are following:

Here,

In the above model, the activity of Esp1 is dependent on the amount of Pds1. Thus the model can resist against the over-expression of *ESP1* up to more than 256 fold. However, the model cannot reproduce the reduction of the upper limit of *ESP1* in *clb2Δ* cell. And the *PDS1* over-expression makes the upper limit of *ESP1* reduced (Figure S4D). The upper limit of *ESP1* is reduced down to 20 fold, when *PDS1* is over-expressed (50 fold), which is inconsistent with the experimental observation in Figure 3D.

**3-3. Esp1 phosphorylation model**

In Xenopus oocyte and human, it is known that CDK inhibits separase (homolog of Esp1) activity with the direct phosphorylation [4]. In the budding yeast Esp1, there are putative CDK phosphorylation sites ([Thr/Ser]-Pro) conserved among other yeast species (Thr-1012, Ser-1025, Thr-1032; Saccharmyces Genome Database, http://www.yeastgenome.org/). We thus modified Chen’s model so that Clb2 inhibited the Esp1 activity via its phosphorylation (Figure S6A).

Changes made from Chen’s model are following:

Sister chromosome separation occurs when

increases above 0.1.

Here, , and G indicates Goldbeter function.

In this model, the upper limit of *ESP1* is more than 256 fold (Figure S6B). In contract, the upper limit of the phosphorylation-negative form of Esp1 (*ESP1-AAA*) is very low (<2). We thus tested this hypothesis by the gTOW experiment with *ESP1-AAA* in vivo, and found the upper limit >100 (Figure 6C). This suggests that this model is incorrect.

**3-4. Pds1 stabilization model**

It is reported that there are two additional CDK phosphorylation sites on Pds1 (Thr-27 and Ser-71), which function to prevent degradation of Pds1 by APC-Cdc20 [5]. We thus modified Chen’s model according to this information (Figure S7A).

Changes made from Chen’s model are following:

Here,

, and G indicates Goldbeter function.

In this model, Pds1 is in large excess of Esp1 (Pds1:Esp1 is 112:1 on average during the cell cycle) and is quickly phosphorylated by Clb2. Parameters for the Pds1 degradation needed to be increased from Chen’s model so that the large amount of Pds1 is degraded effectively. The phosphorylated form of Pds1 is resistant against the Cdc20-dependent degradation. The FEAR activity (i.e. PPX) is constant to simplify the prediction.

In this model, the upper limit of *ESP1* is increased up to 135 fold (Figure S7C), and the upper limit of *ESP1* is highly reduced (<20) when the phosphorylation of Pds1 is disrupted (*clb2Δ* and *pds1-2A* model). Although the upper limit of *ESP1* in *pds1-2A* strain was about 80 (Figure 6C), we set up the parameters (*k’’_kp,pds_, k’’’_kp,pds_, and k’’_d,pds_*; each represents phosphorylation activities of Pds1 by Clb5 and Clb2, degradation activity of Pds1 by Cdc20) to be <20 in the model because there seems to be unknown mechanism in which Clb2 confers the cellular robustness against *ESP1* overexpression (see main text). The effect of Pds1 phosphorylation on Thr-27 and Ser-71 by Clb2 on the upper limit of *ESP1* can be altered by changing the parameters (*k’’_kp,pds_* = 0.8*, k’’’_kp,pds_* = 0.8*, and k’’_d,pds_* = 0.5).

Abundance of Pds1 in the wild type and the *pds1-2A* mutant of this model are not so much different during most of the cell cycle (Pds1:Esp1 is 112:1 in the wild type model and is 1:73 in the *pds1-2A* model on average during the cell cycle) as described in Holt et al. 2008. However, Pds1 is degraded earlier in metaphase in the *pds1-2A* model than wild type model, which causes the imbalance between Pds1 and Esp1 upon *ESP1* overexpression. The imbalance leads to precocious activation of *ESP1* and the cell cycle halt (Figure S8).

In the final model, we also modified Cdc14-Net1 regulation to increase the upper limit of *NET1*:

Here, , and other parameters followed the value defined in Chen’s model.

**4. References**

1. Chen KC, Calzone L, Csikasz-Nagy A, Cross FR, Novak B, et al. (2004) Integrative analysis of cell cycle control in budding yeast. Mol Biol Cell 15: 3841-3862.

2. Queralt E, Lehane C, Novak B, Uhlmann F (2006) Downregulation of PP2A(Cdc55) phosphatase by separase initiates mitotic exit in budding yeast. Cell 125: 719-732.

3. Agarwal R, Cohen-Fix O (2002) Phosphorylation of the mitotic regulator Pds1/securin by Cdc28 is required for efficient nuclear localization of Esp1/separase. Genes Dev 16: 1371-1382.

4. Uhlmann F (2003) Chromosome cohesion and separation: from men and molecules. Curr Biol 13: R104-114.

5. Holt LJ, Krutchinsky AN, Morgan DO (2008) Positive feedback sharpens the anaphase switch. Nature 454: 353-357.
